# Upregulation of a novel eukaryotic translation initiation factor 5A (eIF5A) in dengue 2 virus-infected mosquito cells

**DOI:** 10.1186/1743-422X-7-214

**Published:** 2010-09-07

**Authors:** Yu-Tzu Shih, Chao-Fu Yang, Wei-June Chen

**Affiliations:** 1 Graduate Institute of Biomedical Sciences, College of Medicine, Chang Gung University, Kwei-San, Tao-Yuan 33332, Taiwan; 2 Department of Public Health and Parasitology, College of Medicine, Chang Gung University, Kwei-San, Tao-Yuan 33332, Taiwan

## Abstract

**Background:**

Dengue virus, a mosquito-borne flavivirus, is the etiological agent of dengue fever, dengue hemorrhagic fever, and dengue shock syndrome. It generally induces apoptosis in mammalian cells, but frequently results in persistent infection in mosquito cells. That mechanism remains to be explored. In turn, a genomic survey through subtractive hybridization (PCR-select cDNA subtraction) was conducted in order to find gene(s) that may play a role in interactions between the virus and its host cells.

**Results:**

Through this technique, we identified a novel eukaryotic translation initiation factor 5A (eIF5A) which is upregulated in *Aedes albopictus*-derived C6/36 cells infected by the type 2 dengue (Den-2) virus. The full-length of the identified eIF5A gene consisted of 1498 bp of nucleotides with a 41.39% G+C content, and it possessed a higher similarity and shorter evolutionary distance with insects than with other organisms. Upregulation of eIF5A in response to Den-2 virus infection was validated at both the RNA and protein levels. This phenomenon was also observed by confocal microscopy. In addition, cell death obviously occurred when eIF5A activity was inhibited in C6/36 cells even when they were infected by the virus. However, viral multiplication was not obviously affected in infected C6/36 cells when eIF5A activity was reduced.

**Conclusions:**

Taken together, we postulated that eIF5A plays a role in preventing mosquito cells from death in response to Den-2 viral infection, thus facilitating continued viral growth and potential persistent infection in mosquito cells. It would be worthwhile to further investigate how its downstream factors or cofactors contribute to this phenomenon of dengue infection.

## Background

The dengue virus, one of the flaviviruses, contains ~11 kilobase (kb) single-stranded, positive-sense genomic RNA [1]. Within host cells, viral RNA directly translates into a single polyprotein that is subsequently cleaved into three structural proteins and seven nonstructural proteins [2]. The process is carried out by the combined action of host proteases and a trypsin-like viral NS2B/NS3 serine protease [3].

The dengue virus is transmitted between humans by mosquitoes, implying that both mammalian and mosquito cells are susceptible to the virus [4]. Mammalian cells with dengue virus infection usually end up undergoing apoptosis due to shutdown of protein synthesis in the host cell [5]. However, dengue and other arboviruses frequently occur in mosquito cells without causing obvious deleterious effects [6,7], implying that specific host factors are critically involved in such regulation.

Hypothetically, viruses invading a host cell redirect cellular processes to meet the needs of viral propagation [8], leading to the induction of novel changes in gene expressions; this was reported in human umbilical vein endothelial cells infected with dengue virus [9]. The change in a host cell's protein-making machinery was also confirmed after infection by the dengue virus [10]. In turn, the path to maturation for the dengue virus may depend on the cell type, leading to unique characteristics of the virus.

Through the method of polymerase chain reaction (PCR)-select complementary (c)DNA subtraction, eukaryotic translation initiation factor 5A (eIF5A) was demonstrated to be upregulated at both the messenger (m)RNA and protein levels in C6/36 cells following dengue 2 (Den-2) virus infection [11]. eIF5A, formerly called eIF-4D, was first isolated from immature red blood cells [12], is an acidic protein with a molecular mass of 17~21 kDa, and is relatively conserved from yeast to humans [13]. It is the only protein in nature known to contain the unusual amino acid, hypusine [*N^ε^*-(4-amino-2-hydroxybutyl) lysine], derived from a modification of lysine by spermidine [14].

The eIF5A protein was originally considered to be a translation initiation factor based on its *in vitro *activity of stimulating the formation of methionyl-puromycin, a dipeptide analogue, used in a model system to study the formation of the first peptide bond and to transiently attach to the ribosome in the course of initiation of eukaryotic cellular protein synthesis [15]. However, its role in translation seems controversial since its deletion in yeast leads to only a slight decrease in total protein synthesis [16]. Further, eIF5A was suggested to function as a nucleocytoplasmic shuttle for specific subsets of mRNAs involved in cell division [17], and its posttranslational modification is important for cell survival as well as proliferation [18]. These functions were observed via stimulation of polyamines (putrescine, spermidine, and spermine), which are transformed to active eIF5A [19]. Herein, eIF5A was demonstrated to be upregulated in response to Den-2 virus infection in C6/36 cells, and its role in association with the survival of infected cells is discussed.

## Results

### *Full-length sequence and phylogenetic analysis of eIF5A derived from *Ae. albopictus

**Figure 1:**
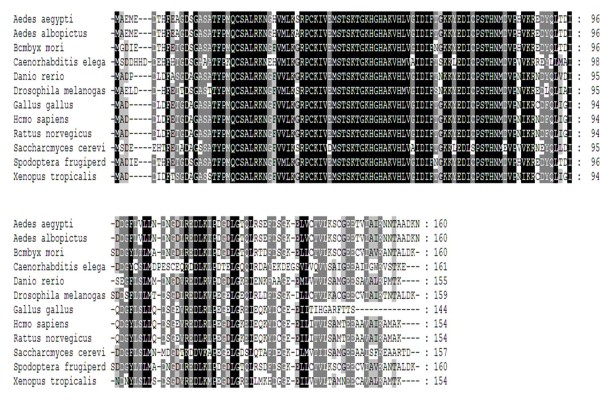
**Alignment of the eIF5A amino acid sequence derived from C6/36 cells with 11 homologous proteins from other organisms**. The black background denotes amino acid residues identical to those in the first line, and gaps are indicated by a dash (-). Accession numbers of listed species: *Aedes albopictus *(EU910137); *Ae. aegypti *(ABF18091); *Bombyx mori *(AAZ15319); *Caenorhabditis elegans *(CAA90247); *Danio rerio *(AAH67190); *Drosophila melanogaster *(AAG17032); *Gallus gallus *(CAG31407); *Homo sapiens *(NP_001961); *Rattus norvegicus *(NP_001028853); *Saccharomyces cerevisiae *(BAA11826); *Spodoptera frugiperda *(AAF13316); and *Xenopus tropicalis *(CAJ83651).

Full-length eIF5A derived from *Ae. albopictus *consists of 1498 bp of nucleotides with a 41.39% G+C content and possesses an 85.8% similarity with that from *Ae. aegypti *(AY433334). The sequence was submitted to GenBank (accession no. EU910137). This genome encoded 160 amino acids, with only a single amino acid difference (S→A) compared to that from *Ae. aegypti *(ABF18091) (Figure 1).

**Figure 2:**
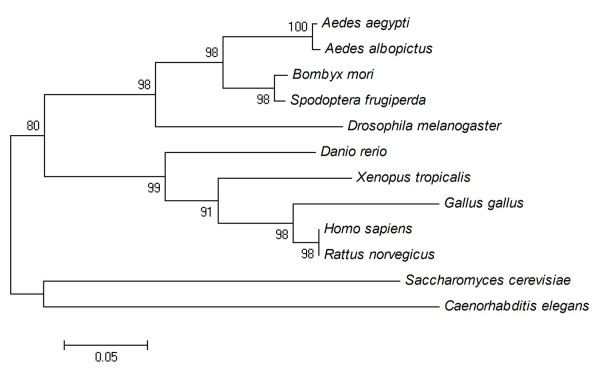
**Neighbor-joining tree of eIF5A identified from 12 species of organisms using protein databases from GenBank**. Numbers on the branches are bootstrap proportions (1000 replicates). See the text for accession numbers.

In a comparison of 12 eIF5A proteins, the one from *Ae. albopictus *shared 99% similarity with that from *Ae. aegypti*, 89% with that from *Bombyx mori *(AAZ15319), 57% with that from *Caenorhabditis elegans *(CAA90247), 69% with that from *Danio rerio *(AAH67190), 80% with that from *Drosophila melanogaster *(AAG17032), 67% with that from *Gallus gallus *(CAG31407), 68% with that from *Homo sapiens *(NP001961), 68% with that from *Rattus norvegicus *(NP001028853), 62% with that from *Saccharomyces cerevisiae *(BAA11826), 90% with that from *Spodoptera frugiperda *(AAF13316), and 68% with that from *Xenopus tropicalis *(CAJ83651). In the phylogenetic tree constructed using the NJ method (Figure 2), at the protein level, the first branch that emerged from the insect group included vertebrates as mentioned above. The bootstrap support for the insect group was 98%; it was as high as 80% for other organisms in the NJ tree. In contrast, eIF5A derived from *Ae. albopictus *was genetically distant from those of fungi (*S. cerevisiae*) and nematodes (*C. elegans*).

### Elevated expression of eIF5A in C6/36 cells infected by the Den-2 virus

**Figure 3:**
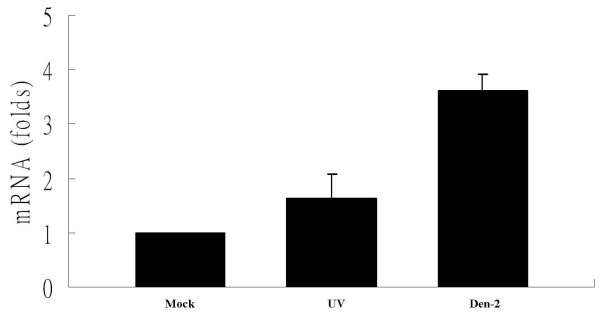
**Validation of the eIF5A gene expression level in C6/36 cells with Den-2 virus infection**. RNAs extracted from C6/36 cells with mock infection (Mock), UV-inactivated Den-2 virus (UV), or intact Den-2 virus (Den-2) at a multiplicity of infection (MOI) of 1 were evaluated by a quantitative real-time RT-PCR assay. The quantitative real-time PCR analysis of eIF5A was monitored and normalized to the expression of 18S, which was used as an internal control. Ratios of the normalized expressions of eIF5A of Den-2-infected cells were relative to that of mock-infected cells. The results showed that the expression of eIF5A was significantly higher in the group with Den-2 virus infection (*p *< 0.05), but not in those inoculated with UV-inactivated Den-2 virus (*p *> 0.05).

**Figure 4:**
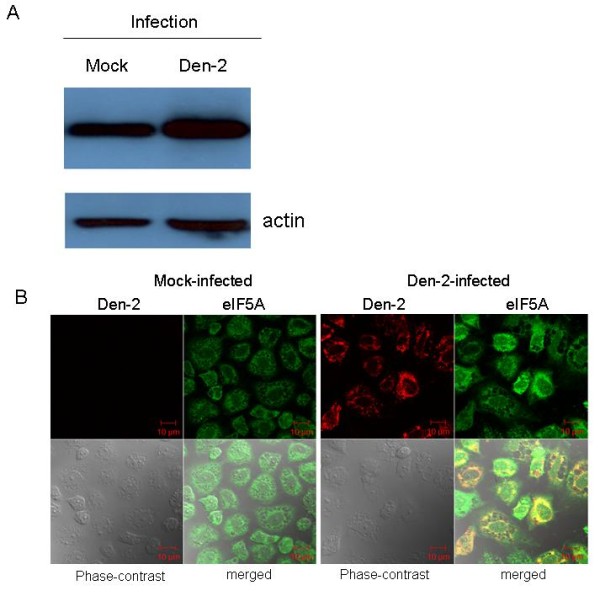
**Upregulation of eIF5A in C6/36 cells infected by Den-2 virus for 24 h**. eIF5A was shown to have increased in expression, according to the results of Western blotting, in response to Den-2 virus infection at 24 h post-infection (hpi) (A). With confocal microscopy, expression of eIF5A (green) was shown to be upregulated in Den-2 virus (red)-infected cells compared to that of the mock infection (B).

Expression of eIF5A in C6/36 cells was analyzed following Den-2 virus and UV-inactivated Den-2 virus infection. C6/36 cells were infected with either the Den-2 virus or a UV-inactivated Den-2 virus at an MOI of 1. At 24 h, cells were collected for RNA extraction. Significant upregulation of eIF5A was only observed in C6/36 cells after infection by intact Den-2 virus as detected by a quantitative real-time PCR. Den-2 virus infection induced a 3-fold (3.60 ± 0.30) increase in eIF5A (for comparison with the mock; Student's *t*-test; *p *> 0.05), whereas the inactivated Den-2 virus infection only induced a 1.63-fold (1.63 ± 0.44) increase (Student's *t-*test; *p *< 0.05) (Figure 3). Enhanced expression of eIF5A at the protein level was measured by Western blotting (Figure 4A). Using double-staining with specific antibodies to compare images under laser scanning confocal microscopy, the expression of eIF5A was obviously enhanced in virus-infected C6/36 cells at 24 hpi compared to mock-infected cells in which lighter expression of the endogenous protein was shown (Figure 4B). Co-localization of eIF5A and dengue proteins was shown in certain areas of infected C6/36 cells (Figure 4B).

### Association of eIF5A with the survival of infected C6/36 cells

**Figure 5:**
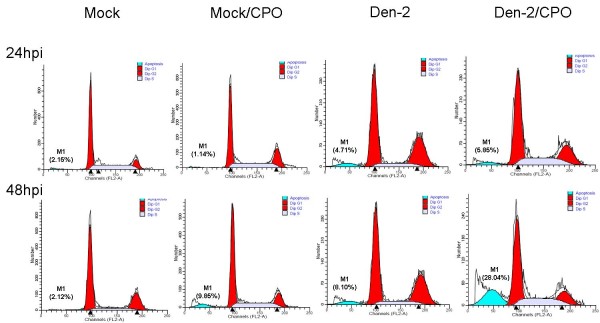
**Effects of eIF5A on the survival of C6/36 cells with Den-2 virus infection**. C6/36 cells were incubated with the Den-2 virus for 1 h at a multiplicity of infection (MOI) of 1, and then treated with ciclopirox olamine (CPO, 10 μM). At 24 and 48 h, cells were fixed and stained with propidium iodide for a flow cytometric analysis. Cells in the sub-G_0_/G_1 _phase are marked as M1, and the rate of cell death is shown in parentheses on each graph. Representative data of the experiments are shown.

Cell death was measured at 24 and 48 hpi using the method of PI staining. With mock infection (without CPO treatment) in C6/36 cells, the cell death rates were 2.15% and 2.12%, respectively; the rates did not evidently change even when cells were treated with CPO (1.14% and 9.85%, respectively). When cells were infected with the Den-2 virus (without CPO treatment), the cell death rate slightly increased to 4.71% and 8.10% at 24 and 48 hpi, respectively. In the group with Den-2 virus infection plus CPO treatment, the cell death rate slightly increased to 5.85% at 24 hpi, but rapidly to 28.04% at 48 hpi (Figure 5).

### Effects of the eIF5A on propagation of the dengue virus

**Figure 6:**
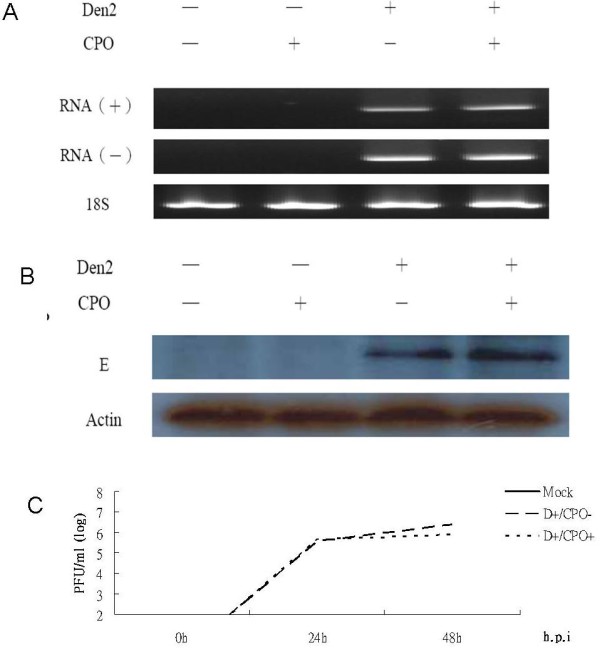
**Inhibition of eIF5A via treatment with CPO showed no effect on propagation of C6/36 cells inoculated with Den-2 virus for 24 h**. (A) Demonstration of viral replication using RT-PCR to amplify fragments of the E gene from extracted positive- (+) or negative (-)-strand viral RNA of infected cells. (B) Detection of dengue E protein with an anti-E monoclonal antibody via Western blot analysis in infected cells. (C) Growth of the Den-2 virus in C6/36 cells with or without inhibition at different times after infection.

After treatment of C6/36 cells with CPO for 24 h, both viral RNA and proteins were examined to evaluate the effect of eIF5A on the growth of Den-2 virus. Total RNA harvested from C6/36 cells was evaluated with the primer pair (D2EL and D2ER) to detect the amplification of positive- and negative-sense viral RNAs. The results showed that viral RNA was detected in infected cells with and without treatment with CPO although they did not quantitatively differ (Figure 6A). At the protein level, they did not show a quantitative difference either (Figure 6B). In addition, virus production was not obviously affected by CPO treatment before 24 hpi, but slightly decreased in the period between 24 and 48 hpi (Figure 6C).

## Discussion

The dengue virus is transmitted by mosquitoes between vertebrate hosts in nature [4], reflecting its ability to grow in both humans and mosquitoes. This way of transmission reveals an important event: the vector must be compatible for virus amplification or can even become persistently infected without causing tissue or cell damage; which is hypothetically regulated by specific genes upregulated by viral stimulation [11]. We recently found that eIF5A is upregulated in C6/36 cells with Den-2 infection, suggesting that this gene may have specific functions in mosquito cells infected with Den-2 virus. This implies that eIF5A may play a role different from that in mammalian cells. Because C6/36 cells are upregulated to express eIF5A only by intact, not by UV-inactivated, Den-2 virus, its overexpression is supposedly regulated when endocytosis is completed. Levels of the virus in infected cells treated with CPO compared to non-treated cells did not significantly differ. This indicated that induction of eIF5A may just prolong the survival of infected cells, providing an environment beneficial for viral growth.

Phylogenetic analyses using the amino acid frequencies of conserved proteins are free from the drastic bias of the genomic G+C content and may provide a robust estimation of early divergences in the evolution of eukaryotes [20]. As shown in the phylogenetic tree constructed using the NJ method, eIF5A was suggested to have a closer evolutionary relationship and common functions due to the short genetic distance between species compared. The high sequence conservation of eIF5A across species suggests that the protein has an important common physiological role.

Unlike mammalian cells, mosquito cells are usually susceptible to Den-2, but infection does not result in cell death. Based on a cell-cycle analysis, both mock- and Den-2-infected C6/36 cells tended to remain in the S phase, a point at which mosquito cells are good at replication, protein synthesis, and assembly of the virus [21]. It would be interesting to see if inhibiting eIF5A in infected mammalian cells also induces G_1 _arrest as opposed to uninfected cells. This experiment could be crucial in revealing the significance of data in this report. In fact, reduction of eIF5A was previously described to induce G_1 _arrest in mammalian HeLa cells [22]. In C6/36 cells not treated with CPO, the death rate slightly increased in infected cells, indicating that the Den-2 virus may naturally cause a low-level death rate in mosquito cells. On the other hand, eIF5A upregulation induced by the virus actually helped mosquito cells survive the lethal effects of the virus by allowing successful progression through the cell cycle. Although the functions of eIF5A are still being debated, it was reported to play roles in cell proliferation, cell viability, and cell-cycle progression. In addition to proliferation and cellular protein synthesis [15], genetic and pharmacological studies provided evidence that eIF-5A is essential for cell survival [23]. Although the expression of the eIF5A protein is normally low [24], the Den-2 virus likely induces eIF5A overexpression in C6/36 cells, which is advantageous for cells' adaptation to viral infection without deleterious effects.

Taken together, we postulate that the Den-2 virus may stimulate the overexpression of eIF5A, which facilitates a reduction in cell death in infected C6/36 cells. This actually produces an advantage of continuing replication by the virus in mosquito cells although it might not be involved in directly promoting virus replication.

## Methods

### Virus and cell culture

The Den-2 virus (New Guinea C strain) was propagated in *Aedes albopictus*-derived C6/36 cells, which were cultured in minimal essential medium (MEM; GIBCO™, Invitrogen, Carlsbad, CA, USA) supplemented with 10% fetal bovine serum (FBS), 2% non-essential amino acids, 2 g/ml Hepes (Sigma, St. Louis, MO, USA), 2.2 g/ml sodium bicarbonate (NaHCO_3_), and 0.4% antibiotic-antimycotic at 28°C in a closed system. The virus was titrated as described below in baby hamster kidney (BHK)-21 cells, which were maintained in MEM containing 10% FBS, 2% non-essential amino acids, 2.2 g/ml sodium bicarbonate (NaHCO_3_), and 0.4% antibiotic-antimycotic (GIBCO™, Invitrogen) at 37°C in a 5% CO_2 _atmosphere. Viruses produced in cultured cells were titrated by a plaque assay as described previously [11].

### Cell infection

C6/36 cells (~1 × 10^7 ^cells/tube) were harvested and centrifuged at 3000 rpm and 4°C for 10 min. After removing the medium, the Den-2 viral suspension or medium (mock infection as the control) was added to the tubes at a multiplicity of infection (MOI) of 1 for incubation at 25°C for 1 h with gentle agitation every 15 min. Then the viral suspension was removed by centrifugation, and pelleted cells were seeded and incubated at 25°C.

### RNA extraction and reverse-transcription polymerase chain reaction (RT-PCR)

The procedures of RNA extraction and RT-PCR were performed as described previously [11]. In brief, total RNA was isolated from both mock- and Den-2 virus-infected C6/36 cells using the Trizol reagent (Invitrogen). Complementary (c)DNA was prepared from extracted total RNA following instructions provided by the SMART™ PCR cDNA synthesis kit (Clontech, Mountain View, CA, USA).

### Real-time PCR

cDNA from infected (with active or UV-inactivated virions) or uninfected (mock) cells was used to validate the expression of eIF5A using the primers GCCCATCCACTCACAACATG (forward) and TCGATGTCAGTGAGCTGGTAGTC (reverse), designed from the sequence of the cloned eIF5A described above. The thermal cycling conditions and presentation of results followed a previous description [11].

### Determination of the full-length sequence of eIF5A

Determination of the full-length sequence of eIF5A followed an approach described elsewhere [25]. Extracted total RNA was used to synthesize a fragment of eIF5A with Oligo dT and the primer derived from selected clones of eIF5A (eIF5AL: 5'-TATTTGCCCATCCACTCACA). The products were then cloned into the pGEM-T vector to subsequently sequence the 3'-end of the gene. The 5'-end of *Ae. albopictus eIF5A *was obtained using a 5'RACE system (Invitrogen) according to the manufacturer's protocol. In brief, the extracted total RNA was first treated with 1 U/μl DNase (Promega, Madison, WI, USA) to remove the genomic DNA, from which 5'-end cDNA was generated with gene-specific primer (GSP)-1, 5'-CGATGCCAACCAGATGTACC-3', and Superscript II™ RT (Invitrogen). dCTP was added to the tail of the 5'-end cDNA using terminal deoxynucleotidyl transferase, and then the dCTP-tailed cDNA was amplified by a PCR with GSP-2, 5'-GTGTTTACCGGTCTTGGAGG-3', and universal primers provided by the manufacturer of the kit. The resultant PCR products were then cloned into the pGEM-T vector (Promega) for nucleotide sequencing. The obtained sequence was used to compare ESTs derived from both *Ae. aegypti *and *Armigeres subalbatus *[26].

### Phylogenetic analysis

The similarity of the eIF5A coding sequence derived from *Ae. albopictus*-derived C6/36 cells was compared, using the basic local alignment search tool [27] in the BLAST network service (National Center for Biotechnology Information, Bethesda, MD, USA), against those from selected species (see "Results") in the database. All sequences were aligned using the default parameters of CLUSTAL X [28] and edited by Genedoc software [29]; from this, a phylogenetic analysis using an unrooted tree constructed with the distance-based Neighbor-joining (NJ) method was carried out with MEGA4 [30]. One thousand bootstrap replications were performed. Other parameters used the default option.

### UV inactivation of the Den-2 virus

The method followed a previous description [11]. Briefly, a viral suspension was exposed to a UV lamp (254 nm; 120 mJ/cm2) for 30 min. The efficacy of viral inactivation was examined by a real-time RT-PCR and plaque assay. Expression of the eIF5A gene in UV-inactivated Den-2 viral-infected C6/36 cells was assayed by a real-time RT-PCR as described above.

### Detection of viral RNA synthesis

Synthesis of viral RNA including positive and negative strands was detected by an RT-PCR as described before [31]. Viral RNA was extracted from C6/36 cells inoculated with a combination of Den-2 virus (at an MOI of 1) and CPO. Inoculated cells were harvested at 24 h post-infection (hpi) to detect RNA synthesis through amplification of a gene fragment by RT-PCR. The primer pair (D2EL: TAACACCACAGAGTTCCATC and D2ER: TAAACTTTCCTGTGCACATA) was used to detect newly synthesized positive-strand RNA. The primers used to detect negative-strand RNA was the complementary counterparts of the above primer pair. The PCR product was identified as 429 bp of an amplified cDNA fragment by running on a 2% (w/v) agarose gel.

### Confocal microscopy

About 2 × 10^6 ^C6/36 cells were plated in 6-well culture plates for 24 h. A Den-2 virus suspension was added to each well and allowed to be adsorbed for 1 h, and then the cells were was incubated for another 24 h. Cells were fixed with 4% paraformaldehyde and subsequently treated with 0.1% Triton X-100 for 2 min to increase the permeability. Primary antibodies including a rabbit anti-eIF5A antibody (1: 8000 in dilution) and a mouse anti-Den-2 antibody (1: 100 in dilution), followed by secondary antibodies of Alexa Fluor^® ^488-conjugated goat anti-rabbit IgG (Invitrogen) and rhodamine-conjugated goat anti-mouse immunoglobulin G (IgG) (Chemicon International, Billerica, MA, USA), were used to respectively detect eIF5A (in green) and the Den-2 (in red) virus. 4'-6-Diamidino-2-phenylindole (DAPI) which presented as blue was used as an indicator of cell nuclei. Prepared specimens were observed under a laser scanning confocal microscope (Zeiss LSM 510, Vertrieb, Germany).

### Effects of eIF5A on cell death measured with propidium iodide (PI) nucleic acid staining

C6/36 cells (~2 × 10^6 ^cells/tube) were collected and infected with the Den-2 virus at an MOI of 1. After 1 h of absorption, cells were treated with 10 μM ciclopirox olamine (CPO, Sigma) to inhibit the function of eIF5A, while treatment with dimethyl sulfoxide (DMSO; the solvent used with CPO) was used as the control. At 48 hpi, cells were harvested and centrifuged at 1000 rpm and 4°C for 5 min. After the suspension was removed, the cell pellet was fixed with ice-cold 70% ethanol in a -20°C freezer for at least 1 h. Cells were centrifuged again at 1500 rpm and 4°C for 5 min, and washed with PBS after the fixative solution had been discarded. These cells were treated with 0.5% Triton X-100 and 0.05% RNase A (Sigma) in PBS for 1 h at 37°C. After a final centrifugation, pelleted cells were stained with 50 μg/ml PI (Sigma) in PBS at 37°C for 20 min and stored at 4°C in the dark. The cellular DNA content was measured using ModFit LT software vers. 3.0 (Verity Software House, Topsham, ME, USA) with a FASCAN flow cytometer (BD Biosciences, San Jose, CA, USA).

### Western blotting

To detect viral proteins, C6/36 cells were infected with the Den-2 virus at an MOI of 1. At 24 hpi, cells were harvested and washed with PBS three times. Approximately 2 × 10^6 ^cells were pelleted and lysed with 100 μl RIPA lysis buffer (50 mM Tris Cl (pH 7.4), 150 mM NaCl, 1% NP-40, 1 mM EDTA, and a protease inhibitor cocktail) at -80°C overnight. After centrifugation at 14,000 rpm for 10 min at 4°C, supernatants were boiled in 2× sample buffer (8% sodium dodecylsulfate (SDS), 1 M Tris (pH 6.8), 40% glycerol, and 0.001 bromophenol blue) for 10 min; these were subsequently resolved by SDS-polyacrylamide gel electrophoresis (PAGE) and transferred to an Immobilon™-P transfer membrane (Millipore, Billerica, MA, USA). Membranes were soaked in 5% skim milk in a TBS-T solution (0.242% Tris-base, 2.924% NaCl, and 0.1% Tween 20; pH 7.5) at room temperature for 1 h. Membranes were then washed with the TBS-T solution three times. For viral protein detection, membranes were probed with an anti-Den-2 viral E protein antibody at room temperature for 1 h; for eIF5A detection, membranes were probed with an anti-eIF5A antibody (both of which were prepared by our lab). A goat anti-rabbit IgG-horseradish peroxidase (HRP)-conjugated antibody (Perkin-Elmer™ Life Sciences, Boston, MA, USA) was subsequently added to the membranes and incubated for 1 h at room temperature after the membranes had been washed with TBS-T. After the membranes were washed again, band profiles were visualized by a reaction after application of Western Lighting^® ^Chemiluminescence Reagent Plus (Perkin-Elmer™ Life Science, Waltham, MA, USA) and exposure to Kodak BioMax XAR film (Eastman Kodak, Rochester, NY, USA).

### Statistical analysis

Comparisons between two means were analyzed by Student's *t*-test at a significance level of 5%.

## Competing interests

The authors declare that they have no competing interests.

## Authors' contributions

YTS carried out all the experiments and analyzed results. CFY helped to perform confocal microscopy. WJC designed the study and wrote the manuscript. All authors read and approved the final manuscript.
